# Understanding Vaccine Hesitancy in U.S. Prisons: Perspectives from a Statewide Survey of Incarcerated People

**DOI:** 10.3390/vaccines12060600

**Published:** 2024-05-31

**Authors:** Emily Greberman, Erin Michelle Turner Kerrison, Aaron Chalfin, Jordan M. Hyatt

**Affiliations:** 1 School of Criminal Justice, Rutgers University, Newark, NJ 07102, USA; 2 School of Social Welfare, University of California, Berkeley, CA 94720, USA; kerrison@berkeley.edu; 3 Department of Criminology, University of Pennsylvania, Philadelphia, PA 19104, USA; achalfin@sas.upenn.edu; 4 Department of Criminology & Justice Studies, Center for Public Policy, Drexel University, Philadelphia, PA 19104, USA; jmh498@drexel.edu

**Keywords:** vaccine, incarceration, attitudes, trust, acceptance, hesitancy, policy

## Abstract

Much of the American response to the COVID-19 pandemic was characterized by a divergence between general public opinion and public health policy. With little attention paid to individuals incarcerated during this time, there is limited direct evidence regarding how incarcerated people perceived efforts to mediate the harms of COVID-19. Prisons operate as a microcosm of society in many ways but they also face unique public health challenges. This study examines vaccine hesitancy—and acceptance—among a sample of individuals incarcerated within adult prisons in Pennsylvania. Using administrative records as well as rich attitudinal data from a survey of the incarcerated population, this study identifies a variety of social and historical factors that are—and are not—associated with an incarcerated person’s willingness to receive the COVID-19 vaccine. Our findings highlight vaccination challenges unique to the carceral context and offer policy recommendations to improve trust in credible health messengers and health service provision for this often overlooked but vulnerable population.

## 1. Introduction

The COVID-19 pandemic brought about massive disruptions to economic and social life in the United States. This includes a nearly unprecedented spike in unemployment [1] and homicides [2], and notably politicized, acrimonious public debates about the safety of COVID-19 vaccines [3,4] and the wisdom of public as well as private sector vaccine mandates [5,6]. Soon after the development of novel vaccines, public agencies, from the U.S. Centers for Disease Control and Prevention to local governments across the country, began to mobilize public health resources to garner support for vaccination. Six months after the vaccines were approved for use by the U.S. Food and Drug Administration, 53.7% of Americans had received at least one dose of an approved COVID-19 vaccine [7]. By 2021, the share of the vaccinated population exceeded 80% [7]. Vaccine acceptance, though, has been strikingly heterogeneous, with fragmented research noting differences in acceptance by region and type of residence [8], race [9], education [10], political partisanship, and varying religiosity [11].

Interest in vaccines has also varied according to an individual’s risk of dying or suffering serious medical issues as a result of COVID-19. Accordingly, older Americans, as well as those with serious or chronic health conditions that predispose them to higher risk of poor COVID-related health outcomes, were more likely to have received the COVID-19 vaccine than younger and healthier and therefore lower-risk individuals [12,13,14]. Beyond the biologically determined predictors of developing serious complications from the novel coronavirus, Americans’ risk of contracting and dying from COVID-19 has varied according to socioeconomic and contextual factors. Hospital workers, first responders, and essential workers [15,16,17] have faced higher incidences of COVID-19 infection, especially during the first year of the pandemic when vaccines were unavailable. The risks have also been higher for individuals residing in denser living conditions [18,19,20], a fact that may help to explain persistent race gaps in COVID-19 infection and death in the United States.

Consistent with the widespread availability of vaccines and prevailing hesitancy rates, approximately 73% of Americans had received at least one dose of an approved COVID-19 vaccine and 62% of Americans have received at least two doses by the end of 2021 [21]. Among the U.S. population, there were wide divides in vaccination rates along the lines of race, age, and education. Black and Hispanic Americans were less likely to accept the vaccine than White Americans, while Asian Americans had the highest rate of self-reported vaccine acceptance at 80% [9]. The education gap is likewise large, with vaccine acceptance rates reaching 70-88% among Americans with a college or graduate education but only 50-69% among Americans with a high school diploma or less [10,22]. Concerning age, older Americans, who are at far greater risk for serious complications from COVID-19, are considerably more likely to have received the vaccine than their younger counterparts. Other determinants of vaccine acceptance among the general public include prior willingness to receive other vaccines such as the influenza vaccine [23] and a Republican political identity [12,24].

As with the remainder of the U.S. population, once vaccines became widely available, one of the primary barriers to vaccine acceptance in United States prisons has been hesitancy [25]. When comparing vaccine administration for the general public to those who are incarcerated, estimates of the share of Americans who received a vaccine within the first 6 months of rollout (December 2020–May 2021) stand between 41% (two shots) and 50% (one shot) [7]. On the other hand, the nationwide prison record data suggest that approximately 55% of the incarcerated population had received at least one vaccine dose during the same period, indicating that vaccine hesitancy is potentially lower among incarcerated populations [26]. At the same time, vaccination rates have remained stubbornly low. By June 2021, only 10 states had reported that over 70% of their incarcerated population was vaccinated [27,28]. In Pennsylvania, the setting for this research, the share of people incarcerated in prisons who are vaccinated is far higher than in other states. After about one year of vaccine administration within correctional facilities, 73% of incarcerated individuals were fully vaccinated [29].

The resistance is multi-faceted, with individuals in prison (people who have been convicted of a crime and whose criminal sentence was greater than two years, in this jurisdiction) citing concerns with side effects and the long-term safety of vaccine receipt as well as factors that reflect the challenges of the carceral context and larger structural health inequities that are uniquely pronounced in the USA. With most vaccine research highlighting the perceptions of society as a whole or within local community-based subgroups, the attitudes and use of medical services by incarcerated individuals remains relatively overlooked [30]. One way of tackling questions about incarcerated patients’ use of prison-based medical services is to consider who this population identifies as credible messengers about vaccine information.

When studying the extent to which incarcerated people trust vaccines and prison-based healthcare more generally, it is critical to highlight the systemic racist practices that marginalized subjects confront in prison sites. Throughout history, Black individuals have been mistreated and abused through medical experimentation where they have been harmed without consent for exploitative research and financial gains [31]. Within a correctional context where the detained are disproportionately people of color, nonconsenting incarcerated individuals have historically been subject to the unethical testing of drugs and vaccines [32]. Over time, this has understandably resulted in a skepticism towards medical providers. Given the racist history of medical provision practices writ large and within the microcosm of prison sites, and that incarcerated people were originally more likely to accept a vaccine then their counterparts beyond the prison wall, we aim to reconcile these contradictions by exploring who incarcerated individuals trust to be credible messengers for vaccine information. To that end, we consider how race, trust, conviction classification, and other demographic information may be predictive of vaccine hesitancy and acceptance.

Investigating how incarcerated people perceive certain vaccines is imperative to correctional and public health policy. Examples of currently available research has studied the willingness to accept vaccines by pregnant women [23], the immunocompromised [33], and essential workers [24,34], as well as among the general public [35,36,37,38]. Overall, approximately 20–27% of Americans indicate some hesitancy to receive a COVID-19 vaccine [39,40,41]. To date, far less is known about correctional populations (for exceptions, see [42,43]) despite their elevated exposure risk. Across the US, the COVID-19 mortality rate was approximately one-third higher in prisons than it was among the broader U.S. population, with incarcerated individuals experiencing a rate of 39 deaths per 100,000 people in prisons, compared to 29 deaths per 100,000 among the general population [44]. Death rates have been especially high at larger facilities that house more than 1000 incarcerated people [45]. Out of the largest 100 clusters of COVID outbreaks, 90 of them occurred within US prisons [46] (Pennsylvania prisons, in particular, experienced positivity rates that outpaced those of the general population. One facility that houses primarily elderly and sick individuals reported a positive infection rate of over 50% between November and December 2020 [47]).

Since incarcerated people live together in close quarters, have little control over their daily interactions, lack access to the types of protective measures that are common in the community, and are unable to socially distance themselves to the same degree as other Americans, they contracted COVID-19 at a rate that was considerably higher than the remainder of the U.S. population [44]. People in prison (individuals who have been convicted of a crime and whose criminal sentence was greater than two years, in this jurisdiction) were also more likely to die from COVID, a fact which may be further explained by their difficulty in quickly accessing high-quality healthcare [48,49] as well as the fact that people in prison are, on average, in worse health than other Americans, especially when controlling for age [18,50,51], which may be exemplary of the history between minority communities who tend to be overrepresented among the carceral population and medical authorities [52,53].

There are several potential reasons for the high rates of COVID-related death among incarcerated people. First, in addition to living close to one another, often sharing a cell with at least one other person, prior research shows correctional populations experience higher co-morbidity prevalence and are less healthy than the general population of the United States, with elevated rates of high-risk health conditions such as diabetes [54], hepatitis [55], and obesity [56]. Second, the higher risks are not limited to incarcerated people; correctional staff have likewise experienced higher rates of COVID-19 infection than the general population [57], which makes sense given their high degree of close contact with incarcerated people and a working context that is characterized by many of the same environmental risk factors. Some of these factors include the constant movement of incarcerated individuals and staff between different cells, prison units, and facilities [58], the lack of sanitation in toilets, showers, and kitchens [59], and even poor ventilation [59], which multiplies the risk for disease transmission.

Several demographic and individual history attributes may correlate with COVID-19 vaccination uptake (or lack thereof), which include age, race, educational attainment, and the most serious offense for which incarcerated people are convicted. Since age is such a strong predictor of serious illness, examining the expanding geriatric population within prisons is especially crucial to understanding COVID-19 death rates. Shortly before incarcerated respondents completed our survey in the early months of 2021, Pennsylvania prisons had already experienced a high volume of deaths from COVID-19, approximately 1,766 cases by December 2020—or one in twenty-one incarcerated people [60,61]. This is a particularly compelling concern in PA, as the state has one of the largest populations of individuals serving life without parole—around 13.4% of the incarcerated population, versus 3.6% nationally [62]. Revisiting minority groups’ experiences with medical providers, in a survey of general population adults in Michigan, Black participants were found to have more medical distrust than other racial groups [63]. Within a prison context, a COVID-19 vaccine hesitancy survey taken by people incarcerated within the Federal Bureau Of Prisons found that 54% of people who did not take the vaccine cited concerns over distrust of the vaccine [64]. Combined with the distrust in correctional employees and healthcare providers [65], the prison environment may exacerbate the already existing distrust people of color have towards medical authorities. However, using it to explain why one does or does not want the COVID-19 vaccine only tells a partial story [63]. Regarding education and literacy, people incarcerated specifically within the Pennsylvania Department of Corrections (where our participants are housed) had an average reading level of mid-8th grade and 24% had not completed high school in 2021 [66]. As educational advancement often coincides with an individual prioritizing and monitoring their health, likely due to better opportunities to regularly maintain it [67], it is important to explore if similar assumptions between education attainment and vaccine acceptance hold for carceral populations.

Nowotny and colleagues [68] used prison-specific factors to understand how individuals may be enabled to use health services. When looking at health service use between people charged with violent crimes versus drug crimes, for example, they found that being an individual with a violent offense increases the odds of using health services than those with drug offenses. They theorized that combined with the inconsistent rates of healthcare use by facility, utilization disparity by conviction offense may suggest differences in the availability of health services based on security level. They also found a positive correlation between sentence length and health services utilization, where every additional year of being incarcerated contributed to 21% higher odds of using health services. The authors also note that this correlation diminishes over time. In sum, the rate of medical service usage greatly varied by facility, thus making it difficult to deduce generalized claims about the use of health services in varying facilities.

Turning to an attitudinal lens that considers trust in vaccine information and its sources, as opposed to the characteristics of the incarcerated individual and their environment, we explore trust in medical information from sources other than traditional health authorities restricted by state authority as an indicator of vaccine uptake. Further unveiling who individuals trust as messengers for vaccine information may demonstrate a need to identify credible health information sources beyond prison staff authorities. Lessard et al. [42] addressed these aspects of vaccine hesitancy in a small-scale survey of people incarcerated in Canadian federal prisons that examines which factors were seen as barriers to receiving a COVID-19 vaccine. They found that 73% (n = 11) of participants intended to take the COVID-19 vaccine, but that perceived misinformation about the vaccine had led to hesitancy.

Related to distrust are other important issues to consider such as the lack of choice and autonomy in prison. Studies looking at the power of choice for incarcerated individuals highlights that perceived afforded choice can correlate with a better perceived quality of life in prison [69]. It is therefore possible that incarcerated individuals view options such as getting a vaccine as an important choice for their own bodily autonomy. Rejecting the vaccine may be an opportunity to increase the sense of agency for the individual [70], as the freedom of choice in a prison environment is rare. However, an important concern is that while they may have the choice to get vaccinated, research shows that some expect to be pressured into receiving it if they expressed hesitancy [42]. As freedom to guide one’s health is varied across prisons, identifying trusted sources of medical information is especially critical for a generally less empowered population.

This paper seeks to fill an important gap in the literature by facilitating a more elaborate understanding of the explanatory power of demographic information alone as drivers of vaccine acceptance among correctional populations, focusing on a large and representative sample of people incarcerated in Pennsylvania state prisons. By investigating incarcerated persons’ attitudes towards the vaccine, we hope to highlight the relationship between varied trust in sources of medical information, such as medical providers in the facilities, staff, their families, their community, and the government and the likelihood of COVID-19 vaccination. By complementing the existing administrative demographic information with attitudinal data, this paper highlights how demographics such as race, age, and offense type may not provide as full an explanation for why members of an extremely vulnerable population reject or accept a vaccine. As such, we aim to develop a more comprehensive understanding of the reasons behind vaccine hesitancy among incarcerated individuals.

## 2. Data and Methods

We report results from a survey of a sample of people incarcerated within all Pennsylvania state correctional institutions (SCIs, 21 male and 2 female facilities). The survey was rolled out in February 2021 by the Pennsylvania Department of Corrections in coordination with local-level correctional authorities and was administered before the availability of any COVID-19 vaccines [71]. In Pennsylvania prisons, there were 37,485 total incarcerated across the state.

After a period of survey development, incarcerated people at each prison were selected for the survey using stratified random sampling and weighted by facility size. Within each prison, a random sample of incarcerated people, proportionate to the population of that facility relative to the system-wide population, was selected. The survey was administered by DOC staff using portable, secure tablet computers, with the selected individuals permitted to use the tablet to complete the survey in relative privacy before recording their responses, sanitizing the tablet, and returning it to staff. Across all prisons, 2111 incarcerated people were randomly selected to receive the survey. Overall, 1498 surveys were turned in, resulting in a response rate of approximately 70%. Sixty-eight of the participants (4.5%) were female, which is proportionate to the female population in the PA DOC system [66]. Since there were relatively few women in the sample, we do not report results separately by gender. Varying by question, the item-level responses were received from roughly 1100–1300 people.

The survey covered a range of questions. First, incarcerated individuals were asked a series of basic demographic questions including their age, race, and gender, and the type of crime that led to their current prison term. They were also asked to indicate their self-reported health status and their vaccination history, emphasizing two factors. The first is their experience receiving a prior influenza shot and the second is how frequently they have received vaccines in the past. Finally, individuals were asked a series of attitudinal questions about their trust in social, governmental, and medical institutions. Attitudinal response types used a five-point Likert scale ranging from ’strongly agree’ to ’strongly disagree’. For ease of interpretation, Likert scale variables which were used to elicit a respondent’s vaccine history and attitudinal variables were converted to binary responses to reflect strongly held preferences. We grouped “strongly agree” and “agree” responses together and “neither agree nor disagree”, “disagree”, and “strongly disagree” together to maximize interpretability and in recognition that Likert scales do not contain cardinal information. The wording was changed for the two health-related variables. Originally asked on a Likert scale, the variable “You were vaccinated as an adult” was originally posed in the survey, “As an adult you were vaccinated against at least one disease” and was made binary. For “You were in good health before the pandemic”, the survey said, “Rate your overall physical health before the pandemic” with options ranging from great, good, average, poor, and terrible. “Great” and “good” were grouped together, and average–terrible were grouped to then create the binary variable. The variable, “You get the Flu vaccine each year” was posed the same in the survey and followed the same binary process.

The results proceed with a descriptive analysis of the survey data before turning to a series of logistic regression models which isolate the importance of a given set of factors, controlling for other predictors. In all models, the outcome variable of interest is a binary measure of interest in vaccine acceptance that is based on the following question: “If made available to you for free, would you take a vaccine for COVID-19?”

Using logistic regression, we regress interest in vaccine acceptance on a series of predictor variables to identify the strongest predictors of vaccine acceptance. Included in this regression are racial demographics (with White as the leave-out group, derived from the Department’s administrative dataset), education (with having less than a high school diploma as the leave-out group, self-reported), age (with under 50 years old as the leave-out group, administrative data), and the most serious and recent offense type for which a respondent was sentenced to custody (with property crimes as the leave-out group, derived from respondent self-report). We then repeat the same exercise separately according to the incarcerated person’s self-reported race to test whether the set of predictors varies by race. We ran models of the following general form:

(1) $$\ln(\frac{{ACCEPTANCE}_{i}\;}{{(1-ACCEPTANCE}_{i})})\;=\;\alpha \;+\;X_{i^{\prime }}\;\beta$$

We include a variable for the incarcerated individuals’ most serious commitment charge (reference category = Property Crimes) and several measures of prior vaccine acceptance. We likewise include binary variables for trust in the following types of individuals: family, community leaders, medical providers, government, and correctional staff. Finally, we include information about an incarcerated persons’ self-reported health status and prior take-up of other types of vaccines.

## 3. Results

Table 1 reports summary statistics derived from the survey. With respect to race, 41% of the respondents identify as White, 43% identify as Black, and 12% identify as Hispanic. A total of 74% of participants are under 50 years old, and the majority of those who filled out of survey have at least a high school diploma (68%). At the time of this study, the larger DOC racial demographics of individuals incarcerated within the PA DOC were similar, with 44% White, 46% Black, 9% Hispanic [66]. To better align with the PA DOC age range scale, we also report that when adjusted to a range of over and under 40 years old (instead of above and below 50), 50% of the DOC population as of 2021 were younger than 40 and 50% were 40 or older [72]. In our sample, 48% of participants were younger than 40 and 52% were 40 or older. Education levels represented here also mirror that of the DOC population, showing that 24% had less than a high school diploma [66]. A plurality of respondents (44%) were serving time for Part 1 offenses as defined by the Uniform Crime Reporting program. These consist of individuals serving time for murder (18%), robbery (9%), aggravated assault (8%), and rape or sexual assault (7%); this is in line with the DOC reporting that 48% of incarcerated individuals are serving time for Part 1 offenses. A total of 11% were serving time for a drug crime, 5% for gun charges, 3% for attempted crimes, and 6% for property offenses, such as burglary, theft, arson, and motor-vehicle theft (7%). A total of 33% of respondents either did not disclose their charge or provided a response that could not be categorized because this question was a self-reported item; therefore, individual crime types may be underreported and underrepresented. A large majority of the sample (83%) indicated that they had received some sort of vaccine in the past, but only 46% indicated receiving a flu vaccine each year. While it cannot be calculated how much of the general population maintains their yearly shot, over the past five years, the annual percentage vaccinated from 2017 to 2021 has ranged from 40 to 55%, naturally raising over time, likely due to the pandemic [73]. These similar uptake rates echo the voluntary nature of getting vaccinated for both populations. With respect to what groups incarcerated people trust to tell them the truth about the vaccine, over three-quarters report they trust their family, 60% trust community leaders and medical staff, and half say they trust correctional staff. In contrast, just one in five respondents trust other incarcerated people to tell them the truth about the vaccine. Overall, most respondents (87%) indicated that they trusted some subset of people to provide them with accurate information.

To provide a sense of how coefficients change with the inclusion of additional covariates, we begin with a simple model that contains only demographic variables and the incarcerated persons’ current commitment charge. We then add attitudinal predictors and measures of baseline health and prior vaccine history. Finally, we re-run (1) separately by race to test whether the predictors of vaccine acceptance vary according to a respondent’s race. In all regressions, we report White standard errors that are robust to arbitrary heteroskedasticity in the regressors.

We begin our analysis with Table 2, which presents coefficients and robust standard errors from a logistic regression of vaccine acceptance on demographic predictors as well as dummy variables for an individual’s current commitment charge.

Compared to White incarcerated people, Black individuals had 26% lower odds of indicating that they would accept the vaccine than White incarcerated people (*p <* 0.01). On the other hand, Hispanic individuals’ willingness to receive the vaccine was not significantly different from that of the White population. With respect to age, incarcerated people above the age of 50 had 47 percent higher odds of accepting the vaccine than younger incarcerated individuals. Concerning the commitment charge, when compared to property crimes (the reference group), people who reported being convicted for murder or rape/sexual assault were more likely to accept the vaccine than those charged with burglary or theft. This follows the findings of Nowotny et al. [68]. However, given that there are too few degrees of freedom to make any empirically driven discernable recommendations based on offense types, it would be interesting to measure whether sentence length motivates vaccine acceptance. No significant differences are apparent for other commitment charges.

In Table 3, we add attitudinal predictors, measures of self-reported health, and prior vaccine acceptance to the model and estimate the vaccination willingness results separately by the individual’s race (columns 2–4). Referring to column 1, we see that when attitudinal variables and health-based factors are added to the model, Black incarcerated people no longer have lower odds of accepting the vaccine. The coefficient reverses and suggests that, if anything, Black individuals who are incarcerated are more likely to accept the vaccine once other relevant factors are controlled for. Instead, the primary predictors for vaccine acceptance are whether an incarcerated person trusts the facility’s medical providers, the government, and community leaders. These findings highlight that while race is typically seen as among the most salient predictors of vaccine acceptance, it is a black box. Using rich attitudinal information, we unpack that black box and find evidence suggesting that racial differences in vaccine hesitancy are explained by differences in trust in national and community institutions as opposed to some other mutable or immutable difference between racial groups. Interestingly, controlling for other predictors, an incarcerated person’s self-reported health status is not predictive of vaccine hesitancy.

Turning to columns (2)–(4), we report race-specific findings and note that the predictors of vaccine acceptance—especially the attitudinal predictors—vary considerably by race/ethnic group. Among White and Black incarcerated people, holding trust in the prisons’ medical providers and government are among the most salient positive attitudinal predictors of uptake. On the other hand, among Hispanic incarcerated people, trust in community leaders is the strongest predictor of hypothetical vaccine uptake. For Hispanic incarcerated people, trust in medical providers is a weak predictor at best and the predictive value of trust in government is imprecisely estimated. Across all groups, prior vaccine history is a strong predictor of hypothetical acceptance of the COVID-19 vaccine.

## 4. Discussion

We report results from one of the first large-scale surveys of vaccine acceptance among a sample of incarcerated individuals in the United States. We find that a considerable majority—70% of the sample—indicated a desire to receive the COVID-19 vaccine when it becomes available to them. Given the demography of Pennsylvania incarcerated people who are disproportionately Black and Hispanic and have low levels of education—characteristics that are typically used in estimates of vaccine hesitancy in the U.S. population generally—this sample offers a high rate of projected vaccine acceptance. We also observed that 13% of the sample indicate that they do not trust anyone to tell them the truth about the vaccine. Given that 87% of participants do trust someone to tell them the truth about the vaccine in some capacity, this suggests that there may be ways to increase vaccine acceptance by tapping into the most credible messengers. Additional research should explore why 13% of the incarcerated population do not trust anyone to tell them about the vaccine and how it might compare to the general population’s response to a similar statement.

Descriptive statistics reveal familiar cleavages in vaccine acceptance along race, education, and age lines. Once attitudinal factors are accounted for, isolated demographic factors no longer solely predict vaccine uptake, highlighting the complex and intertwined nature of racial identity and attitudes towards medical developments. In particular, vaccine acceptance appears to be a function of *trust*, whether that is trust in government, correctional authorities, medical providers, or community leaders. Among Black and White incarcerated individuals, trust in government and medical providers was most pivotal, whereas trust in community leaders was not uniquely predictive. Among Hispanic incarcerated people, the opposite is true. Trust in community leaders is most predictive of vaccine acceptance, while trust in medical providers and government provides a far less predictive signal. Among incarcerated people of all races, prior vaccine history was a strong predictor of COVID-19 vaccine acceptance, following the basis of Lessard et al.’s [42] study.

Our analysis reveals that Black individuals were less likely to be interested in receiving a vaccine than non-Black individuals. Likewise, older individuals were more likely to be interested in receiving the vaccine than younger individuals. However, attitudinal factors play a key role in explaining these familiar demographic patterns in vaccine hesitancy. Individuals who trust community leaders and medical providers to be truthful about the safety of vaccines are more likely to accept vaccines. Once measures of social trust are accounted for, the explanatory power of demographic factors weakens considerably and the race gap in vaccine acceptance becomes small and statistically insignificant. For Hispanic individuals who are incarcerated, trust in information from community leaders is the most important driver of vaccine acceptance, whereas, for Black and White incarcerated individuals, it is trust in medical providers. Therefore, perhaps there may be a way (or there already is a way) to bridge the divide between medical providers, people of color, and trust. Understanding and acknowledging the historical abuse of said minority groups may be a crucial component to including a legitimately perceived messenger for health-related information—acknowledging that people of diverse racial backgrounds may look to different institutions for vaccine information.

While the pandemic continues to linger and diseases such as influenza, RSV, and monkeypox (now referred to as mpox) continue to proliferate, certain actions may increase vaccine acceptance and strengthen preventative healthcare within prisons. First is the need to invest in improving the relationship between incarcerated people and medical staff within correctional facilities. First, providers could be better educated about structural competency and the systemic socioeconomic experiences of people of color navigating healthcare [74,75,76]. Williams and Rucker [77] suggest creating initiatives to train medical professionals with awareness of their historical experiences, as well as making the entrance to being a healthcare provider more accessible for people from minority backgrounds. Having more people of color within healthcare organizations can be beneficial in several ways, such as encouraging empathy, being a part of strategic planning, and creating a greater self-awareness for other providers [78]. To foster a greater sense of trust, being able to report negative experiences within healthcare settings may also be incredibly important. Noah [79] recommends anonymous reporting systems to detect possible systemic patterns of biased treatment and decision making. In a more proactive approach, providers can engage in training programs that teach about cultural nuances and body language, working within communities to better understand the implementation and effectiveness of a given initiative, and cultivating partnerships with local organizations that are respected and trusted by said community [78]. Other priorities to improve trust for incarcerated people are for them to receive appropriate medical screenings, form prosocial relationships with medical staff, access trauma-informed care, and see an increased solution-based response to health-related grievances [80]. This increase in higher-quality contact may help form the basis for greater trust in healthcare institutions in the future.

Lastly, prison leadership might also bring in trusted community leaders—or other credible messengers—from outside the prison community to evangelize support for vaccine acceptance, specifically, or the provision of healthcare, more generally. For example, an effective implementation could integrate health-focused educational programming run by community leaders inside prisons. In doing so, this may minimize long-standing, negative perceptions of healthcare and vaccines, and maximize acceptance and trust in future public health crises. This lack of trust likely exists far before the individual enters prison [81]; therefore, implementing outreach within communities to promote awareness for preventative health measures would be extremely beneficial on a larger scale. We recognize that bringing people into the facilities may also present new exposure and transmission risks. Thus, we propose another alternative way to introduce health-positive programming, and autonomous access to mainstream health information within facilities may emerge through the use of mixed contemporary media. These information sources are imperfect, but they do provide sources of information that are alternative to the covariates observed in our study.

Concerning future research, given the paucity of survey data on vaccine hesitancy among correctional populations, more research is sorely needed. It is a limitation of this research that the survey was administered before any side effects of the vaccines were known. Therefore, their responses may have been different if they knew the possible risks, or lack thereof. Due to these factors, it would be important to examine how vaccine acceptance and uptake may vary when provided information on side effects. Thinking globally about the spread of communicable illnesses in correctional settings, research must study not only people incarcerated but also correctional staff, which is another vulnerable population that is exposed to disparate public health risks and varied access to mainstream evidence-based information about vaccines and health-promoting programming. More of this work could focus not only on the initial hesitancy to receive vaccines by incarcerated people but—given the high eventual rates of vaccination among incarcerated—also on how quickly these incarcerated people ended up becoming vaccinated and which factors were most pivotal in convincing the later adopters, such as information about vaccine side effects and the actual risks of navigating complications. To avoid another dangerous lag of urgent vaccination rollouts, policymakers, politicians, and healthcare providers must work with prison systems to support standardizing and elevating medical care for this population, setting aside social stigma and politicalized debates. Including these various parties with differing experiences and knowledge can only help to make these preventative efforts more efficient.

## 5. Conclusions

This research emphasizes the importance of measuring how vaccine administration in prison is shaped not only by structural challenges, but also by a vulnerable patient population’s attitudes regarding the credibility of medical establishments and their ambassadors. While prisons might provide access to vaccinations, various attitudinal and perceptional factors may complicate accepting said preventative measures. If more is done to build trust between incarcerated individuals, security staff, and medical staff, incarcerated people may be more receptive to vaccine offerings and recommendations. Highlighting the role of trust as an important factor in medical care more generally, special attention needs to be paid to learning and understanding why certain communities or groups may or may not be trusting of certain medical authorities or organizations. Addressing these barriers may necessitate the inclusion of community members or other people incarcerated individuals deemed as credible messengers. Identifying who individuals trust to tell them the truth about vaccines would provide better guidance for effective awareness, education, and ideally, acceptance. Improving trust in the staff and public officials, as well as the inclusion of community members, could culminate in a well-rounded approach to educating individuals on the importance of healthcare and why the preventative nature of vaccines is a vital element of carceral safety. Vaccinations are a public health intervention that effectively prevents or limits the mass spread of disease. As communities face future epidemics, further understanding the justification for vaccine hesitancy by incarcerated individuals and staff during the COVID-19 pandemic may be a life-saving matter.

## Figures and Tables

**Table 1:** Summary statistics.

Variable	Percentage
Would take the Covid vaccine	71%
Race	
White	41%
Black	43%
Hispanic	12%
Other	4%
Offense Type	
Aggravated Assault	8%
Property Crimes	8%
Possession of Weapon	5%
Drug Crimes	11%
Murder	21%
Rape/Sexual Assault	7%
Robbery	9%
Other Crime	31%
Age Group	
Under 50 years old	74%
50+ years old	26%
Education Degree Obtained	
Less Than High School Diploma	20%
High School Diploma	68%
Advanced Degree	12%
Messengers of Vaccine Information	
You trust your family…	78%
You trust staff in this facility…	49%
You trust medical providers in this facility…	59%
You trust the government…	46%
You trust leaders in your community…	60%
You trust other incarcerated people…	21%
You don’t trust anyone…	13%
Health History	
You were vaccinated as an adult	83%
You get the Flu vaccine each year	47%
You were in good health before the pandemic	79%

Note: Table presents summary statistics for the dependent variable (row 1) and each of the predictor variables used in subsequent regressions. Full statement for attitudinal factors is, “You trust ___ to tell you the truth about the vaccine”, and “You don’t trust anyone to tell you the truth about the vaccine.” All variables under “Messengers of Vaccine Info” were answered on Likert scales.

**Table 2:** Logistic odds ratios for demographics.

Dependent Binary: “I Would Take the COVID Vaccine”	All Population
Black	0.738 **
	(0.110)
Hispanic	0.813
	(0.173)
Other	0.543 **
	(0.169)
High School Diploma	0.880
	(0.147)
Advanced Degree	0.940
	(0.231)
50+ years old	2.420 ***
	(0.416)
Aggravated Assault	1.200
	(0.366)
Possession of Weapon	1.171
	(0.400)
Drug Crimes	1.009
	(0.282)
Murder	1.645 *
	(0.439)
Rape/Sexual Assault	2.664 ***
	(0.997)
Robbery	1.051
	(0.308)
Other Crime	1.322
	(0.336)
Constant	1.981 ***
	(0.509)
Sample’s Vaccine Acceptance	0.706
Observations	1246
Log Likelihood	−727.898
Akaike Inf. Crit	1483.795

Note: Table presents coefficients and odds ratios (in parentheses) from a series of least squares regression models of a binary measure of vaccine acceptance on a series of predictor variables. The sample’s vaccine acceptance is the reported mean. *** *p* < 0.001; ** *p* < 0.01; * *p* < 0.05.

**Table 3:** Logistic odds ratios for all covariates.

Logistic Odds Ratios	All Population	Black	Hispanic	White
	(1)	(2)	(3)	(4)
Black	1.272			
	(0.239)			
Hispanic	0.955			
	(0.255)			
Other	0.719			
	(0.291)			
High School Diploma	0.778	0.713	0.508	0.970
	(0.163)	(0.559)	(0.756)	(0.526)
Advanced Degree	0.922	1.059	9.067	0.890
	(0.289)	(0.798)	(13.758)	(0.782)
50+ years old	1.474 *	0.991	4.851	2.056 **
	(0.317)	(0.726)	(6.338)	(0.958)
Aggravated Assault	1.174	0.717	5.003	1.014
	(0.459)	(0.491)	(6.933)	(0.518)
Possession of Weapon	1.072	0.716	1.277	1.256
	(0.459)	(0.680)	(1972.755)	(0.697)
Drug Crime	1.047	0.841	1.899	0.978
	(0.370)	(0.602)	(2.887)	(0.559)
Murder	1.731	1.371	5.992	1.153
	(0.595)	(0.953)	(8.040)	(0.479)
Rape/Sexual Assault	1.685	1.272	10,985,524.00	1.052
	(0.770)	(0.438)	(7,384,069)	(0.359)
Robbery	1.012	0.908	5.379	0.534
	(0.380)	(0.263)	(3.542)	(0.165)
Other Crime	1.207	0.568	7.640	1.122
	(0.394)	(0.178)	(5.287)	(0.364)
You trust staff in this facility…	1.244	1.322	3.267 *	1.129
	(0.269)	(0.417)	(2.188)	(0.372)
You trust leaders in your community…	1.342	1.391	3.917 **	1.106
	(0.254)	(0.461)	(2.721)	(0.366)
You trust your family…	1.153	1.390	0.760	1.103
	(0.231)	(0.185)	(0.276)	(0.151)
You trust the medical providers…	1.893 ***	2.278 ***	1.012	1.685 *
	(0.381)	(0.245)	(0.306)	(0.225)
You trust the government…	2.510 ***	2.067 **	3.551 *	3.252 ***
	(0.514)	(0.610)	(0.2.073)	(0.887)
You don’t trust anyone...	0.839 **	0.814	1.366	0.802
	(0.075)	(0.278)	(0.958)	(0.245)
You were vaccinated as an adult	1.624 ***	1.624 ***	2.508 ***	1.469 ***
	(0.012)	(1.236)	(3.792)	(0.825)
You get a vaccine each year	3.034 ***	4.075 ***	2.539	2.644 ***
	(0.538)	(1.304)	(1.533)	(0.975)
You were in good health before the pandemic	0.766	0.632	0.498	0.979
	(0.158)	(0.315)	(0.916)	(0.480)
Constant	0.369 **	0.671	0.110	0.409
	(0.147)	(0.511)	(0.166)	(0.230)
Observations	1142	482	134	478
Log Likelihood	−505.543	−218.405	−50.026	−201.255
Akaike Inf. Crit.	1057.085	476.809	140.051	442.510

Note: Table 3 presents coefficients and odds ratios (in parentheses) from a series of least squares regression models of a binary measure of vaccine acceptance on a series of predictor variables. Column (1) reports estimates for the entire sample. In columns (2)–(4), we report results for the sub-sample of respondents who are Black (column 2), Hispanic (column 3) and White (column 4). Full statement for attitudinal factors is, “You trust ___ to tell you the truth about the vaccine”, and “You don’t trust anyone to tell you the truth about the vaccine.” All variables after “Other Crime” were answered on Likert scales. *** *p* < 0.001; ** *p* < 0.01; * *p* < 0.05.

## Data Availability

Data sharing or archiving is not allowed under the agreement with the agency that provided it due to the sensitive nature of the records.
